# Very Large Salivary Calculus

**Published:** 1879-01

**Authors:** 


### VERY LARGE SALIVARY CALCULUS.

Dr. Freudenberg relates the case of a gentlemen who consulted him on account of a tumor under the tongue, which gave him only moderate uneasiness, and did not cause any increase of the normal saliva. It dated back six months, and had been since then continually on the increase. On inspection a salivary concretion was found to be projecting to the extent of a centimetre, and by a little manipulation the calculus was dislodged by mere pressure, springing out at last as if from an elastic ring. In length the concretion measured 3.5 centimetres, the diameter of its base measured 1.4 centimetre. It consisted of phosphate and carbonate of lime, and an albuminous substance, the phosphate constituting an unusually large proportion. The calculus occupied the right sublingual gland.—*Berlin Klin. Woch.—Medical Times and Gazette.*

